# *Phellinus linteus* Mycelium Alleviates Myocardial Ischemia-Reperfusion Injury through Autophagic Regulation

**DOI:** 10.3389/fphar.2017.00175

**Published:** 2017-04-04

**Authors:** Hsing-Hui Su, Ya-Chun Chu, Jiuan-Miaw Liao, Yi-Hsin Wang, Ming-Shiou Jan, Chia-Wei Lin, Chiu-Yeh Wu, Chin-Yin Tseng, Jiin-Cherng Yen, Shiang-Suo Huang

**Affiliations:** ^1^Institute of Pharmacology, National Yang-Ming UniversityTaipei, Taiwan; ^2^Department of Anesthesiology, Taipei Veterans General Hospital, National Yang-Ming UniversityTaipei, Taiwan; ^3^Department of Physiology, Chung Shan Medical University and Chung Shan Medical University HospitalTaichung, Taiwan; ^4^Institute of Medicine, Chung Shan Medical UniversityTaichung, Taiwan; ^5^Institute of Biochemistry, Microbiology and Immunology, Medical College, Chung Shan Medical UniversityTaichung, Taiwan; ^6^Division of Allergy, Immunology, and Rheumatology, Chung Shan Medical University HospitalTaichung, Taiwan; ^7^Immunology Research Center, Chung Shan Medical UniversityTaichung, Taiwan; ^8^Department of Culinary Arts, Chung Chou University of Science and TechnologyChanghua, Taiwan; ^9^Department of Health Food, Chung Chou University of Science and TechnologyChanghua, Taiwan; ^10^Department of Pharmacy, Chung Shan Medical University HospitalTaichung, Taiwan; ^11^Department of Pharmacology, Chung Shan Medical UniversityTaichung, Taiwan

**Keywords:** *Phellinus linteus*, cardioprotection, myocardial ischemia-reperfusion, autophagy, apoptosis

## Abstract

The incidence of myocardial ischemia-reperfusion (IR) injury is rapidly increasing around the world and this disease is a major contributor to global morbidity and mortality. It is known that regulation of programmed cell death including apoptosis and autophagy reduces the impact of myocardial IR injury. In this study, the cardioprotective effects and underlying mechanisms of *Phellinus linteus* (Berk. and Curt.) Teng, Hymenochaetaceae (PL), a type of medicinal mushroom, were examined in rats subjected to myocardial IR injury. The left main coronary artery of rats was ligated for 1 h and reperfused for 3 h. The arrhythmia levels were monitored during the entire process and the infarct size was evaluated after myocardial IR injury. Furthermore, the expression levels of proteins in apoptotic and autophagic pathways were observed. Pretreatment with PL mycelium (PLM) significantly reduced ventricular arrhythmia and mortality due to myocardial IR injury. PLM also significantly decreased myocardial infarct size and plasma lactate dehydrogenase level after myocardial IR injury. Moreover, PLM administration resulted in decreased caspase 3 and caspase 9 activation and increased Bcl-2/Bax ratio. Phosphorylation level of AMPK was elevated while mTOR level was reduced. Becline-1 and p62 levels decreased. These findings suggest that PLM is effective in protecting the myocardium against IR injury. The mechanism involves mediation through suppressed pro-apoptotic signaling and regulation of autophagic signaling, including stimulation of AMPK-dependent pathway and inhibition of beclin-1-dependent pathway, resulting in enhancement of protective autophagy and inhibition of excessive autophagy.

## Introduction

Myocardial ischemia is caused by a lack of coronary blood supply to the heart. During myocardial ischemia, a characteristic pattern of metabolic and ultrastructural changes leads to irreversible injury. To limit myocardial infarct size and reduce mortality, early restoration of blood flow to the ischemic myocardium is a common treatment strategy (Schofield et al., 2013). However, the return of blood flow can cause additional cardiac damage and complications, such as paradoxical increases in infarct size and induction of arrhythmia. This is known as myocardial ischemia-reperfusion (IR) injury (Yellon and Hausenloy, 2007; Folino et al., 2013). Incidence of myocardial IR injury is rapidly increasing around the world and this disease is a major contributor to global morbidity and mortality (Hausenloy and Yellon, 2013; Mozaffari et al., 2013). Decreasing the impact of myocardial IR injury is important in the prevention and treatment of ischemic heart disease.

Recent studies have shown that myocardial cell death is the main reason for the poor prognosis of patients with ischemic heart disease. Ischemic and reperfusion injuries to the heart cause various types of cardiomyocyte death such as necrosis, apoptosis, and autophagy (Tian et al., 2013). Among these cell death types, apoptosis (nuclear fragmentation, plasma membrane blabbing, cell shrinkage and loss of mitochondrial membrane potential and integrity) and autophagy (cytoplasmic vacuolization, loss of organelles and accumulation of vacuoles with membrane whorls) are the two principal pathways of programmed cell death. These, may also be important pathways in myocardial IR injury (Eltzschig and Eckle, 2011; Jennings, 2013).

It has been reported that crosstalk exists between apoptosis and autophagy (Maiuri et al., 2007). Activation of autophagy leads to removal of damaged organelles and phagocytic clearance of apoptotic cells (Qu et al., 2007). Autophagy, a cellular process associated with degradation and recycling of damaged organelles and long-lived cytosolic protein, is activated in the heart during normal physiological state and further stimulated under stress conditions such as myocardial IR injury (Mizushima et al., 2008). Under physiologic conditions, autophagy exists at low levels, playing a protective role by degrading damaged mitochondria and protein aggregates. However, excessive autophagy under pathologic conditions can lead to organ dysfunction due to degradation of essential proteins and organelles. These conditions can include IR, nutrient deprivation, infection, hypoxia, and mitochondrial dysfunction (Mizushima et al., 2008). During autophagy, cytoplasmic organelles and proteins, including mitochondria, endoplasmic reticulum (ER), Golgi complex, and endosomes are surrounded and sequestered by isolation membrane and nascent membranes fused at their edges. These form autophagosomes, which subsequently undergo a stepwise maturation process. During this process, there is fusion with lysosomes, resulting in the delivery of cytoplasmic contents to lysosomal components. Finally, there is degradation and recycling (Kang et al., 2011). Defective autophagy can influence cellular functions related to different diseases (Levine and Klionsky, 2004). Cells can recycle extracted amino acids and fatty acids for energy production and sequester damaged organs during autophagy to prevent release of reactive oxygen species (ROS) (Levine and Klionsky, 2004; Sciarretta et al., 2011). However, cell death may be induced by excessive activation (Mizushima et al., 2008; Yu et al., 2010). In brief, insufficient or excessive autophagy is a possible mechanism of disease.

*Phellinus linteus* (Berk. and Curt.) Teng, Hymenochaetaceae (PL), a well-established medicinal mushroom, has been used in Asian countries for centuries to prevent or treat ailments as diverse as hemorrhage, rheumatoid arthritis, gastroenteric dysfunction, diarrhea, and cancers. A number of studies have revealed that PL alleviates septic shock and has antitumor (Kim et al., 2003; Zhu et al., 2007; Park et al., 2010; Konno et al., 2015), immunomodulatory (Song and Park, 2014;Lin et al., 2015; Suabjakyong et al., 2015), anti-angiogenic (Sliva et al., 2008; Lee et al., 2010), and antioxidant properties (Lee et al., 2011, 2015). Moreover, in rats intraperitoneally administered filtrate of PL broth culture, dose-dependent decreases in cortical infarct volume have been demonstrated following permanent focal cerebral ischemia (Suzuki et al., 2011). From these findings, PL may have a beneficial effect on ischemic tissue injury. In addition, hispidin, a phenolic compound from PL, protects H9c2 cardiomyoblast cells and neonatal rat ventricular myocytes against oxidative stress-induced apoptosis. In addition, hispidin scavenges intracellular ROS and increases heme oxygenase-1 and catalase expressions (Kim et al., 2014). There have been no studies to date on the effects of PL mycelium (PLM) on myocardial IR injury *in vivo* or the molecular mechanism involved in such effects. Therefore, we ligated coronary artery in rats for 1 h of ischemia followed by 3 h of reperfusion to evaluate the effects of PLM on myocardial IR injury. Rats were divided into treatment and control groups. Comparisons were carried out between groups. These comparisons included the durations and incidences of ventricular tachycardia (VT) and ventricular fibrillation (VF), as well as infarct size and mortality. We also investigated the cardioprotective effects of PLM on signaling pathways involved in autophagy. In the present study, the aim was to explore the potential of PLM for protecting against myocardial IR injury *in vivo*, as well as the underlying mechanisms.

## Materials and Methods

### Animals

Sprague-Dawley rats (LASCO Co., Charles River Technology, Taipei, Taiwan), each weighing 250–300 g, were used. In this study, based on the Guide for the Care and Use of Laboratory Animals published by the US National Institutes of Health (NIH Publication No. 85–23, revised 1996). The animal study protocol was approved by the Institutional Animal Ethics Committee of Taipei Veterans General Hospital, Taipei, Taiwan (IACUA 2015-241). Animals were housed under conditions of controlled temperature (24 ± 1°C) and humidity (55 ± 5%), as well as 12-h light–dark cycle. Rats were allowed *ad libitum* access to food and water.

### Experimental Design

The rats were randomly assigned to three groups: (1) sham; (2) myocardial IR (control); (3) PLM treatment (PLM). The experimental animals were administered PLM at a dosage of 10^-8^ or 10^-9^ g/kg via jugular vein 15 min prior to left anterior descending artery (LAD) ligation under urethane anesthesia as previously described. The sham and control animals were treated with normal saline. LAD was ligated for 1 h and then reperfused for 3 h. At the end of the experiment, rat hearts were harvested to evaluate infarct size and protein expression levels by triphenyl tetrazolium chloride (TTC) staining and immunoblotting.

### *Phellinus linteus* Mycelium (PLM) Preparation

*Phellinus linteus* mycelium was supplied by Chiu-Yeh WU’s lab (Chuang Chou University of Science and Technology, Changhua County, Taiwan) and prepared as previously described (Wu et al., 2012). *P. linteus*, kindly supplied by Baoji Biotechnology Company Limited, Nantou city, Nantou county, Taiwan, and had been identified by Bioresource Collection and Research Center, Hsinchu, Taiwan, was employed for the production of mycelial biomass and bioactive compounds. The stock culture was inoculated on potato dextrose agar (PDA, Himedia Laboratories, Mumbai, India) slants and incubated at 30°C for 14 days and then stored at 4°C.

To prepare the experimental inoculant, the microorganism was initially grown on PDA plates, and then transferred to the inoculum medium by punching out six pieces of 1 cm^2^ of the agar plate culture with the sterilized cutter. The inoculum culture was incubated in a 300 ml baffled flask containing 100 ml potato dextrose broth (PDB, Himedia Laboratories, Mumbai, India) under 30°C, 100 rpm on a rotary shaker incubator for 24 h. The production culture in a stirred-tank bioreactor (Model FMT-5L, Yuh Chuen Chiou Ind. Co., Ltd., Kaohsiung, Taiwan) for PL was carried out on the fermentation medium compositions (w/v): dextrose 2%, peptone 0.1% and malt extract 2%, and was sterilized at 121°C for 30 min. Fermentations were carried out with 3 L fermentation medium under the following conditions: inoculum ratio 5%, temperature 30°C, aeration rate 1.5 vvm and agitation rate 125 rpm. The cultivation was maintained for 20 days after the inoculum was transferred to the medium. After finishing cultivation, the harvest of mycelial biomass were filtered by filter paper, and then the resulting precipitate was washed repeatedly by ddH_2_O and dried at 50°C to a constant weight. 50 g dried mycelia biomass was extracted by 500 mL ddH_2_O at 121°C for 15 min, cooled down, and filtered, and this step was repeated twice. The filtrate was collected and condensed by lyophilized. The powder was dissolved and dilute to working concentration (10^-8^ and 10^-9^ g/kg) with normal saline.

### Surgical Procedure

Temporary occlusion of the left main coronary artery was carried out to induce myocardial IR injury, as previously described (Huang et al., 2005). Briefly, urethane was used to anesthetize the rats (1.25 g/kg, i.p.). After the trachea was cannulated to provide artificial respiration, polyethylene catheters (PE-50) were placed in the common carotid artery. There was continuous monitoring of heart rate (HR) and arterial blood pressure (BP) via sensors. Data were displayed on a data acquisition unit (MP35) and physiological recorder (BIOPAC Systems, Inc., Goleta, CA, USA). Silver electrodes were attached to the extremities for recording of standard lead-1 ECG.

A respirator for small rodents (Model 131, NEMI, USA) was used for ventilation with room air. Stroke volume was set at 10 ml/kg body weight and stroke rate at 60 strokes/min. Following sectioning of the fourth and fifth ribs, at 2 mm to the left of the sternum, left thoracotomy was carried out to open up the chest. Subsequently, the heart was quickly externalized and inverted. Following placement of 6/0 silk ligature around the left main coronary artery, The animal was allowed 15 min of recovery following repositioning of the heart in the chest. If there was arrhythmia or sustained decrease in BP to less than 70 mmHg following the procedure, the animal was excluded from the study.

### Evaluation of Arrhythmia

Antiarrhythmic effects of PLM during myocardial IR injury were analyzed. Changes in HR, BP, and ECG were simultaneously recorded on a personal computer with waveform analysis software (AcqKnowledge, Biopac System, Goleta, CA, USA) before and during ischemia and reperfusion. Ventricular ectopic activity was evaluated based on Lambeth Convention-recommended diagnostic criteria. Incidences and durations of VT and fibrillation (VF) were determined in both surviving rats and in those that eventually died. The duration of VF was recorded until BP was lower than 15 mmHg in rats that died due to irreversible VF.

### Morphological Analysis

Rats were sacrificed at 4 h post-IR injury for infarct volume analysis (TTC staining) (Bohl et al., 2009). Following removal, the heart was perfused with cold saline solution for 5 min, and flow rate was adjusted to the appropriate range, approximately 3 ml/min. Then, the crown artery was ligated again, followed by perfusion with Evans blue, which stains the remote myocardium but leaves the area at risk (AAR) unstained. Next, the heart was sectioned into standard transverse slices of 2-mm thickness using a heart matrix slicer (Jacobowitz Systems, Zivic-Miller Laboratories Inc., Allison Park, PA, USA). Slices of the heart were stained with vital dye TTC (2%; Sigma), first in the dark at 37°C for 30 min and, then, in 10% formalin at room temperature for 2 days. After scanning of infarcted tissue slices, the weights of tissues were evaluated by distinguishing the blue colored remote myocardium from the AAR and infarct area shown in white on TTC staining assay.

### Estimation of Myocardial Injury

Lactate dehydrogenase (LDH) activity in plasma was measured to evaluate myocardial cellular damage. Following IR injury, arterial blood was collected from the carotid catheter and mixed with heparin (about 100 IU). LDH activity was determined based on a previously described method (Tsai et al., 2004). Following reduction of NAD to NADH by LDH, NADH interacts with a specific probe, resulting in the production of color (max = 450 nm) on colorimetric LDH quantification assay (BioVision, Milpitas, CA, USA).

### Immunoblotting

Left ventricle samples were homogenized with tissue protein extraction reagent (Thermo, Waltham, MA, USA) containing Protease Inhibitor Cocktail (Sigma-Aldrich, St. Louis, MO, USA). Concentrations of proteins were quantified using a protein assay dye reagent (Bio-Rad, Hercules, CA, USA) and bovine serum albumin (BSA) as the standard. Following the mixing with equal volumes of loading buffer [62.5 mM Tris-HCl, pH 6.8, 10% (v/v) glycerol, 2% SDS, 5% (v/v) 2-mercaptoethanol and 0.05% (w/v) bromophenol blue], samples were heated to 95°C for 10 min. They were then separated with SDS-PAGE gel and transferred onto PVDF membranes (GE Healthcare, Chicago, IL, USA). Blocking of membranes was achieved with 1% polyvinylpyrrolidone (PVP) and 0.05% tween 20 in phosphate buffer solution (PBS) at room temperature for 1.5 h, followed by incubation with primary antibodies including anti-Bcl2 (Abcam, Cambridge, MA, USA), anti-Bax (Abcam, Cambridge, MA, USA), anti-caspase 3, anti-caspase 8, anti-caspase 9, and anti-AMPKα and phospho -AMPKα, anti-phosphoinositide 3 kinase (PI3K) p85, anti-phospho PI3K p85, anti-phospho AKT (Cell Signaling, Danvers, MA, USA). In addition, anti-mTOR, anti-AKT (Spring Bioscience, Pleasanton, CA, USA) and phospho -mTOR (Santa Cruz, Dallas, TX, USA); anti-LC3 (Novus Biologicals, Littleton, CO, USA); anti-beclin-1 (Novus Biologicals, Littleton, CO, USA); anti-p62 (Novus Biologicals,, Littleton, CO, USA) and anti-β-actin (Sigma-Aldrich, St. Louis, MO, USA) at 4°C overnight. The membranes were washed three times with PBS containing 0.1% tween-20 (PBST) followed by horse radish peroxidase-conjugated secondary antibodies (1:10,000 dilution) at 37°C for 1 h. The blots were analzyed with enhanced chemiluminescent system following additional PBST washes.

### Statistics

Data are expressed as mean ± standard error of mean (SEM). Differences in IR-induced cardiac infarction and immunoblotting were analyzed. The differences between IR control and PLM-treated groups were ascertained by one-way analysis of variance. The variables were subsequently analyzed to determine if there were significant differences between the groups using Student’s *t*-test. The differences in incidences of arrhythmia and mortality between IR control and PLM-treated groups were analyzed on Chi-square assay. Statistical significance was defined as *p* < 0.05. All tests were two-tailed.

## Results

### Hemodynamic Changes During Coronary Artery Occlusion

**FIGURE 1:**
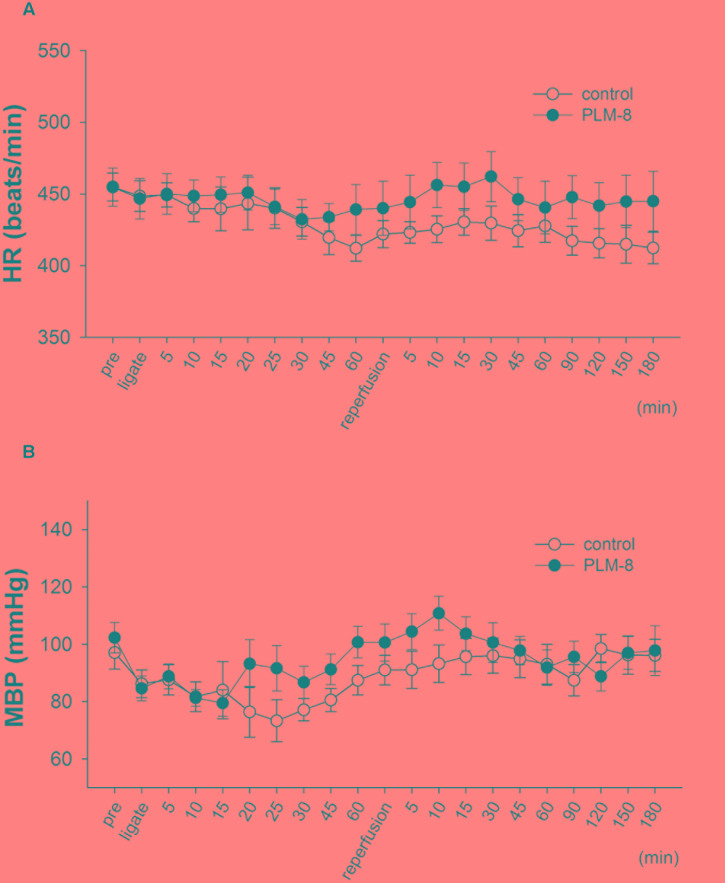
**Lack of hemodynamic changes during coronary artery occlusion and reperfusion in control and PLM-treated groups. (A)** Heart rate change in vehicle (open circle, *n* = 10) and 10^-8^ g/kg (close circle, *n* = 11) PLM-treated groups during myocardial ischemia for 1-h followed by reperfusion for 3-h in rats. **(B)** Mean arterial blood pressure change in vehicle (open circle, *n* = 10) and 10^-8^ g/kg (close circle, *n* = 11) PLM-treated groups during myocardial IR in rats. Each experimental group is comprised of 10 rats. Results are expressed as mean ± SEM. The differences between vehicle and PLM-treated animals were not statistically significant (ANOVA). ‘Pre’ indicates before coronary ligation.

Fifteen minutes after PLM administration via jugular vein injection there were no significant alterations in mean arterial BP or HR of anesthetized rats. In addition, there were no significant hemodynamic changes between vehicle and PLM-treated groups during 1-h myocardial ischemia and 3-h reperfusion periods (**Figures 1A,B**).

### Myocardial IR Induced Rhythm Disturbances

Effects of PLM on anesthetized rats following myocardial IR-elicited arrhythmia are presented in **Table 1**. The incidence and duration of VT were 100% and 30.6 ± 10.8 s during 1 h myocardial ischemia and 3 h reperfusion in vehicle group. Administration of 10^-9^ and 10^-8^ g/kg PLM resulted in a decrease in the incidence of VT by 75%. In addition, the duration of VT significantly decreased in PLM (10^-9^ and 10^-8^ g/kg) treated groups by 4.5 ± 1.6 and 10.8 ± 3.8 s, respectively. The incidence % of VF was 75% in vehicle-treated animals with dose dependent decreases in PLM-treated groups (50 and 12.5% in PLM 10^-9^ and 10^-8^ g/kg, respectively). Administration of PLM also significantly improved the duration of VF. Duration of VF was 41.8 ± 14.8 s in vehicle group, and significantly decreased to 0.5 ± 0.2 s in 10^-8^ g/kg PLM-treated group. VF is the severe type of arrhythmia and it is followed by irreversible death. This phenomenon may imply that PLM decreases myocardial IR-induced arrhythmia and mortality in a dose-dependent manner.

**Table 1:** Effect of *Phellinus linteus* mycelium (PLM) on coronary ischemia 1 h-reperfusion 3 h (IR) induced arrhythmias in anesthetized rats.

			Ventricular tachycardia		Ventricular fibrillation		Mortality
		*n*	Incidence (%)	Duration (s)	Incidence (%)	Duration (s)	(%)
Sham							
	Vehicle	4	–	–	–	–	–
	PLM 10^-7^ g/kg	4	–	–	–	–	–
Operated (IR)							
	Vehicle (Control)	8	100	30.6 ± 10.8	75	41.8 ± 14.8	50
	PLM 10^-9^ g/kg	8	75	4.5 ± 1.6^∗^	50	29.5 ± 10.4	37.5
	PLM 10^-8^ g/kg	8	75	10.8 ± 3.8	12.5^∗^	0.5 ± 0.2^∗^	0^∗^

*Vehicle is in normal saline; n = number of experiments; values for duration of VT and VF are shown as the mean ± S.E.M. ^∗^Statistical difference at the level of P < 0.05 as compared with control.*

### Myocardial Infarct Size

**FIGURE 2:**
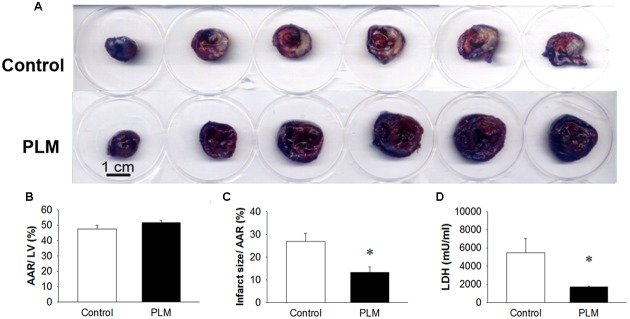
**Ischemia-reperfusion-induced cardiac infarction was rescued by PLM treatment.** Normal saline or PLM 10^-8^ g/kg was administered to rats 15 min before coronal artery ligation. Infarct volumes were determined after 1-h ischemia and 3-h reperfusion. Myocardial infarct size was assessed by Evans blue/2,3,5-triphenyltetrazolium chloride (TTC) double staining. Representative photographs of heart horizontal sections are shown **(A)**. Blue-stained areas are non-ischemic regions; red-stained areas are ischemic-reperfused but viable regions; white areas are infarcted regions. Mean ± SEM of quantitative risk zone infarcted % (ratio of non-blue area to total area) **(B)**, the infarct size/ AAR % (ratio of white area to non-blue area) **(C)**, and plasma lactate dehydrogenase (LDH) activity **(D)** in age-matched control (*n* = 8), and PLM-treated rats (PLM, *n* = 8). ^∗^*P* < 0.05 represents a significant difference between control and PLM-treated groups. Values are expressed as mean ± SEM.

Changes in myocardial infarct size in control rats and rats administered PLM are shown in **Figure 2**. Evans blue colored remote myocardium blue but not the AAR and infarct area, which are shown in white on TTC analysis (**Figure 2A**). There are no significant difference among the vehicle, 10^-9^ and 10^-8^g/kg PLM. AAR/ left vetrcle (LV; %) demonstrated that the IR model is consistent in our study (**Figure 2B**). Infarct size/AAR % was reduced in PLM 10^-8^ g/kg-treated group (28.02 vs. 12.70%, *P* < 0.05) (**Figure 2C**). The acivity of plasma LDH, a marker of tissue damage, was significant decreased in PLM group (5489 ± 3389 vs. 1718 ± 182 mU/ml) (**Figure 2D**). Hence, administration of PLM decreased the impact of myocardial IR injury.

### PLM Attenuates Myocardial IR-Induced Apoptosis

**FIGURE 3:**
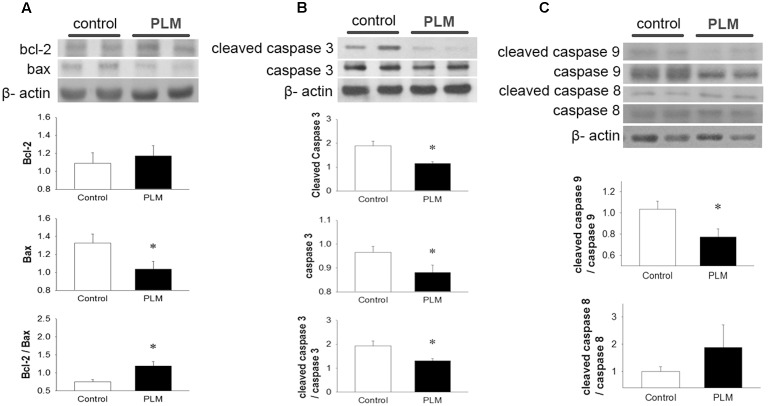
***Phellinus linteus* mycelium deceased apoptosis after IR injury.** Representative Western blots show the levels of Bcl-2, Bax **(A)**, full length and cleaved caspase 3 **(B)** and full length and cleaved caspase 9 and 8 **(C)** in heart tissue. Graphs represent the quantitative differences between control and PLM-treated groups. β-actin was used as a loading control for the blots. ^∗^*P* < 0.05 represents a significant difference between control and PLM-treated groups. Values are expressed as mean ± SEM (*n* = 3).

Apoptotic cell death often occurs in cardiomyocytes after myocardial IR injury. We investigated the expressions of Bcl-2 family proteins and caspase 3, 9, and 8 to assess the anti-apoptotic effects of PLM in rats subjected to myocardial IR injury (**Figure 3A**). Results demonstrated that PLM treatment increases protein expression of Bcl-2, significantly reduces Bax expression and increases ratio of Bcl-2 to Bax in the myocardium after myocardial IR injury when compared with control.

Caspase 3 is a key protein in apoptotic cells, both in extrinsic and intrinsic pathways. Thus, we used it to quantify the apoptotic level in the heart after IR injury (**Figure 3B**). Both the pro-form and active form (cleaved form) of caspase 3 were reduced in PLM-treated group when compared with control. In addition, PLM treatment significantly reduced the ratio of active form to pro-form of caspase 3 when compared with control. In PLM treated group, it was also shown the ratio of active form of caspase 9 was decreased, but active form of caspase 8 did not change significantly (**Figure 3C**). These results indicated that PLM attenuates apoptotic level via intrinsic pathway in IR-injured heart.

### PLM Regulates Myocardial IR-Induced Autophagy

**FIGURE 4:**
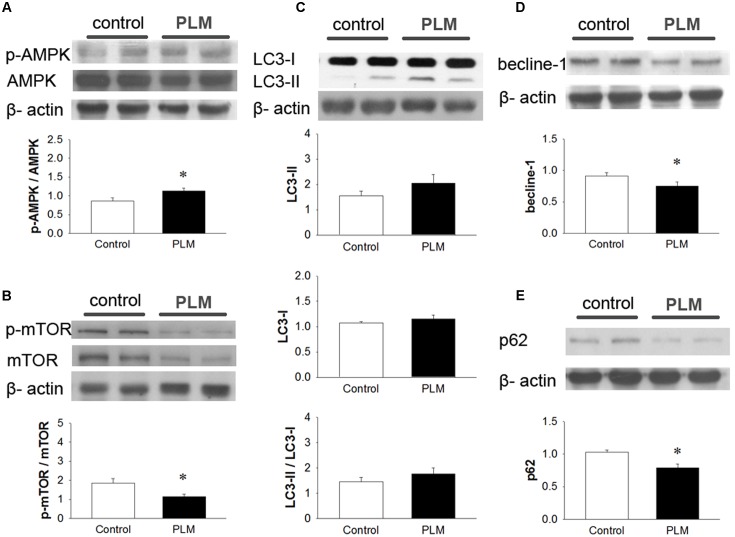
***Phellinus linteus* mycelium increased autophagy after IR injury.** Representative Western blots show the levels of phosphoric AMPK **(A)** phosphoric mTOR **(B)**, LC3I/II ratio **(C)**, beclin-1 **(D)**, and p62 **(E)** in heart tissue. Graphs represent the quantitative differences between the control and PLM-treated groups. β-actin was used as a loading control for the blots. ^∗^*P* < 0.05 represents significant difference between control and PLM-treated groups. Values are expressed as mean ± SEM (*n* = 3).

To examine the role of autophagy in the cardioprotective effect of PLM, we investigated the phosphorylation levels of AMPK and mTOR (p-AMPK and p-mTOR) in left heart ventricle (**Figures 4A,B**). Immunoblotting revealed that treatment with PLM significantly increases the ratio of p-AMPK to AMPK expression and reduces the ratio of p-mTOR to mTOR expression in IR injured myocardium when compared with control.

Regulation of autophagy by mTOR-dependent pathways also could be adjusted by PI3K-AKT pathway (Karki et al., 2016). Hence, we observed the phosphorylation levels of PI3K and AKT (p-PI3K and p-AKT) in left heart ventricle (Supplementary Figures 1A,B). However, the activation of PI3K and AKT had no significantly diffence bteewn control and PLM treated group.

LC3 is widely used to monitor autophagy. LC3-I is the cytosolic form. LC3-II is conjugated with phosphatidylethanolamine (PE) and is present in isolation membranes, autophagosomes, and to a much lesser degree in autolysosomes. In our experiments, when compared with control group, LC3-I did not change in the PLM-treated group. The expression of LC3-II and the ratio of LC3-II to LC3-I increased in PLM-treated group, but this increase did not reach significance (**Figure 4C**). However, beclin-1, a major protein in the nucleation step of autophagy, and p62, a protein targeting specific cargo of autophagy (Rusten and Stenmark, 2010), significantly decreased in PLM-treated group (**Figures 4D,E**). Beclin-1 autophagy induction pathway is regarded as deleterious during IR injury (Ma et al., 2015), and cannot be suppressed by Bcl-2. Bcl-2/beclin-1 interaction is an important checkpoint in autophagic induction due to starvation or treatment with autophagy inducers. The ratio of Bcl-2 to beclin-1 increased in PLM-treated group (1.13 ± 0.15 vs. 1 1.85 ± 0.23, *P* < 0.05). These findings suggested that PLM regulates autophagy through stimulation of AMPK-dependent mechanism and inhibition of beclin-1-dependent mechanism in rats after myocardial IR injury.

## Discussion

**FIGURE 5:**
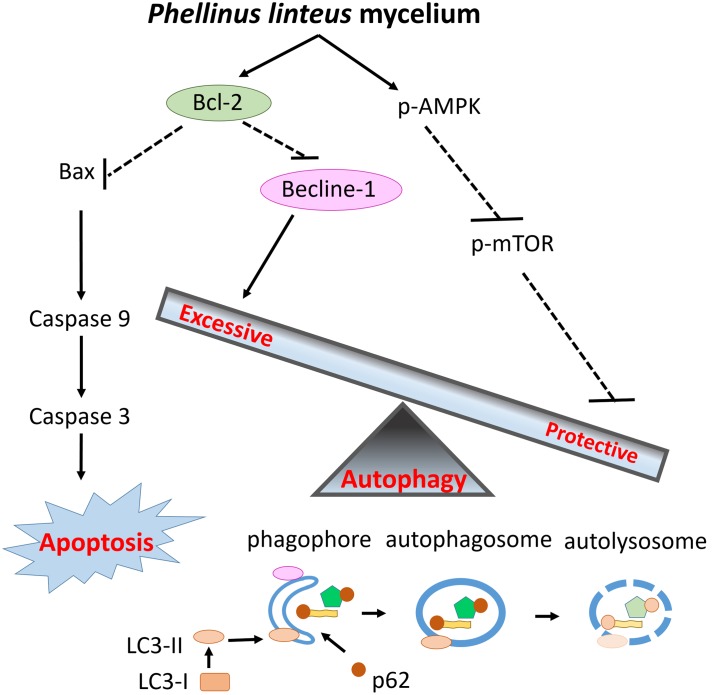
**Schematic diagram of the effects of PLM against myocardial IR injury.** PLM caused apoptotic decrease via increasing ratio of Bcl-2 and Bax. Beclin-1 decreased to attenuate excessive autophagy and the activity of mTOR was downregulated to enhance protective autophagy. Decreased apoptosis and regulation of autophagy play protective roles in myocardial IR injury.

This is the first study to examine the cardioprotective effects of PLM in rats subjected to myocardial IR injury. PLM did not alter the mean arterial BP or HR in studied rats. **Figure 5** summarized the effect of PLM in this study. In the ischemic border zone, the leakage of potassium increased extracellular potassium and caused depolarization of myocytes. This depolarization contributes to electrical heterogeneity of conduction that provide a substrate for reentry, bring about polymorphic VT and/or VF (Koplan and Stevenson, 2009). Administration of PLM effectively suppressed the durations of VT and VF and the incidence of VF at dose of 10^-8^ g/kg, during 1-h myocardial ischemia and 3-h reperfusion period. In addition, myocardial infarction was significantly reduced in PLM-treated group. Compared with control group, LDH activity, which serves as an indicator of cellular damage in plasma, significantly decreased in PLM-treated group during myocardial IR injury, which is consistent with the observation of decreased myocardial infarction. The results of this study support the potential application of PLM as a cardioprotective agent in myocardial IR injury.

Apoptosis, as well as necrosis, often occurs in cardiomyocytes after myocardial IR injury. In the myocardial ischemic core, which is completely deprived of oxygen and nutrients, myocardium dies due to necrosis. However, in the ischemic penumbra, where there is hypoxia after myocardial IR injury, reperfusion accelerates the process of apoptosis compared with permanent occlusion and myocardial cell death occurs via both necrosis and apoptosis (Whelan et al., 2010; Chen-Scarabelli et al., 2014). In this study, we conducted immunoblotting of Bcl2, an antiapoptotic protein, and Bax, a proapoptotic protein. Pro-form and active form (cleaved form) of caspase 3 were also used to assess the effects of PLM on apoptosis. We found that PLM reduced the expression level of Bax, increased the ratio of Bcl-2/Bax and reduced active caspase 3. Furthermore, PLM decreased activation of caspase 9, the intrinsic apoptotic caspase; but not caspase 8, the extrinsic apoptotic caspase. This data meant that PLM reduced the apoptosis relating to mitochondria malfunction. These findings suggest that the inhibition of intrinsic apoptosis contributes to the underlying beneficial effect of PLM.

Several studies have reported that myocardial cell death during IR injury occurs by apoptosis, by necrosis and in association with autophagy (Glick et al., 2010; Whelan et al., 2010; Chen-Scarabelli et al., 2014; Gatica et al., 2015). Autophagy is an important intracellular self- degradation process which is activated by myocardial IR injury. However, whether autophagy plays a beneficial or deleterious role during myocardial IR injury is still unclear (Rifki and Hill, 2012). Autophagy is essential in cellular energy mobilization and homeostasis. However, excessive autophagy leads to progressive consumption of cellular constituents and autophagic cell death. In cardiomyocytes, PT1, AMPK agonist, and 3HOI-BA-01, mTOR inhibitor, profoundly upregulate autophagy in cardiomyocytes after oxygen glucose deprivation/reoxygenation and significantly promote cardiomyocyte survival. In addition, administration of PT1 and 3HOI-BA-01 significantly decreases infarct size in murine myocardial IR injury (Huang et al., 2016). Nevertheless, in adult male Sprague-Dawley rats, ischemic preconditioning that includes three cycles of regional ischemia at 5 min each, with alternating cycle of 5 min reperfusion, inhibits beclin-1-dependent excessive autophagy and diminishes cell death induced by myocardial IR injury (Peng et al., 2013). The classical pathways of autophay are beclin-1-mediated autophagy/apoptosis and AMPK-mTOR-mediated mTOR/autophagy mutual feedback signaling. Excessive beclin-1 protein expression during myocardial IR injury may result in an autophagic imbalance that leads to cell death while induction of autophagy via activation of AMPK is beneficial. Matsui et al. (2007) also reported that autophagy plays distinct roles during myocardial ischemia and IR. Myocardial ischemia-stimulated autophagy through AMPK-dependent mechanism may be protective, whereas IR-stimulated autophagy through beclin-1-dependent, but AMPK-independent, mechanism may be detrimental (Matsui et al., 2007). The activation of AMPK inhibits mTOR-dependent signaling, leading to activation of autophagy. In this study, we found that PLM increases the activity of AMPK and suppresses mTOR activity. These results suggest that PLM increases the activation of AMPK to enhance autophagy by mTOR suppression. Due to the degradation of autophagosomes, beclin-1 and p62 were significantly degraded after 1-h ischemia and 3-h reperfusion injury. These findings indicate that PLM regulates autophagy through stimulation of AMPK-dependent mechanism and inhibition of beclin-1-dependent mechanism in rats after myocardial IR injury.

In others researches, it was reported that mTOR manipulate the autophagy was not only regulated by AMPK but also by PI3K-AKT pathway (Karki et al., 2016). In physiological and pathological conditions, PI3K-AKT pathway was crucial to many aspects of cell growth and survival, and which was initiated by receptor tyrosine kinases (RTK) or G-protein–coupled receptors (GPCRs), which located at the cell surface and activated by many kinds of growth factors. (Pyne and Pyne, 2011; Dienstmann et al., 2014). In this study, the expressions of PI3K and AKT were not influenced significantly by PLM treated (Supplementary Data). It might imply that the effect of mTOR-autophagy by PLM treatment was independent to PI3K-AKT pathway.

Several studies have demonstrated that Bcl-2, an antiapoptotic protein, inhibits beclin-1 via direct binding. This indicates that there is negative regulation of beclin-1-mediated autophagy by Bcl-2, and crosstalk between autophagy and apoptosis (Chen-Scarabelli et al., 2014; Ma et al., 2015). In this study, we found that PLM treatment significantly increases the ratio of Bcl-2 to beclin-1. PLM inhibits apoptosis by upregulation of Bcl-2 expression and downregulation of beclin-1 expression. Therefore, PLM simultaneously inhibits apoptosis and excessive autophagy via the modulation of Bcl-2/beclin-1expression.

In this study, we administrate the PLM, not the fruiting body, before myocardial IR injury. In mycelia of PL, the content of protein, amino acid, total sugar, reducing sugar, polysaccharide and saturated fatty acid were more abundant as compared with the fruiting body (Chen et al., 2016). There are several literatures to reported that polysaccharides might play an important role in the activity of alleviating the myocardial IR injuries (Lu and Zhao, 2010; Zhang et al., 2013). The mycelial culture of PL was be found its polysaccharide might have effect on anti-inflammation, which was believed that it resulted from the structures of the high molecule weight could stimulate the reticulo-endothelial system (Song et al., 1995; Baker et al., 2008). Antioxidant such as hispidin was found in PLM (Chen et al., 2016). Hispidin was identified and could scavenge the free radicals (Lee and Yun, 2007; Jung et al., 2008). The anti-inflammatory and antioxidative effects of PLM could contribute to the cardioprotective effect against IR injury and decrease cell programmed death.

## Conclusion

Our results indicated that PLM attenuates myocardial IR injury. The cardioprotective effect of PLM may be mediated via inhibition of apoptosis and regulation of autophagy, stimulation of AMPK-dependent mechanism and inhibition of beclin-1-dependent mechanism in rats subjected to myocardial IR injury. The findings of this study suggest that the administration of PLM prior to myocardial IR injury is a potential approach for the treatment of IR injury.

## Author Contributions

H-HS and S-SH designed research; H-HS, Y-CC, J-ML, and Y-HW conducted research and analyzed data; C-YW and C-YT prepared the *P. linteus* mycelium; H-HS, J-CY, and S-SH wrote the paper. All authors read and approved the final manuscript.

## Conflict of Interest Statement

The authors declare that the research was conducted in the absence of any commercial or financial relationships that could be construed as a potential conflict of interest.
