# Supplementary material for: Phellinus linteus Mycelium Alleviates Myocardial Ischemia-Reperfusion Injury through Autophagic Regulation
Source: Front Pharmacol. 2017 Apr 4;8:175. doi: 10.3389/fphar.2017.00175 (PMC5378821; doi:10.3389/fphar.2017.00175)
Supplement: Supplementary file 1 [file Data_Sheet_1.pdf]

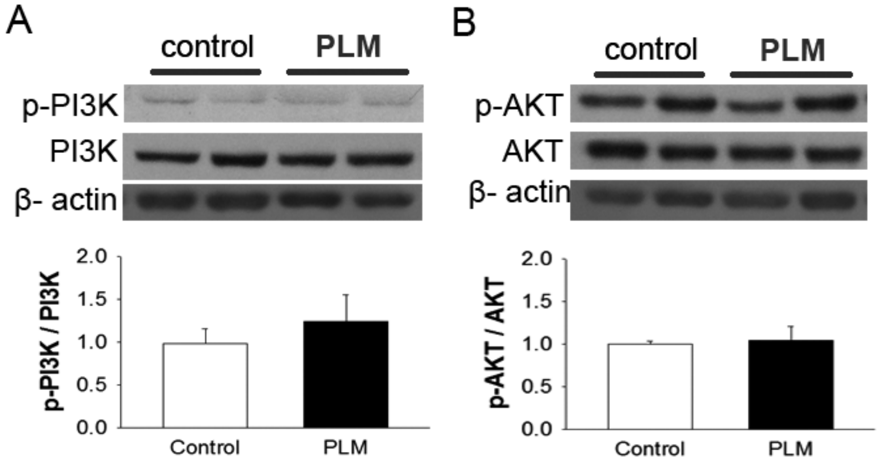

Supplementary Figure 1｜ PLM did not have effects on PI3K and AKT activation after IR injury. Representative Western blots show the levels of phosphoric PI3K (A) and AKT (B) in heart tissue. Graphs represent the quantitative differences between the control and PLM-treated groups. β-actin was used as a loading control for the blots. Values are expressed as mean ± SEM (n=6).
